# The role of CREB3L4 in the proliferation of prostate cancer cells

**DOI:** 10.1038/srep45300

**Published:** 2017-03-24

**Authors:** Tae-Hyun Kim, Joo-Man Park, Mi-Young Kim, Yong-Ho Ahn

**Affiliations:** 1 Department of Biochemistry and Molecular Biology, Yonsei University College of Medicine, Seoul 120-752, Republic of Korea; 2 Brain Korea 21 PLUS Project for Medical Sciences, Yonsei University College of Medicine, Seoul 120-752, Republic of Korea

## Abstract

The incidence of prostate cancer (PC) is growing rapidly throughout the world, in probable association with the adoption of western style diets. Thus, understanding the molecular pathways triggering the development of PC is crucial for both its prevention and treatment. Here, we investigated the role of the metabolism-associated protein, CREB3L4, in the proliferation of PC cells. CREB3L4 was upregulated by the synthetic androgen, R1881, in LNCaP PC cells (an androgen-dependent cell line). Knockdown of *CREB3L4* resulted in decreased androgen-dependent PC cell growth. LNCaP cells transfected with si*CREB3L4* underwent G2/M arrest, with upregulation of the proteins cyclin B1, phospho-CDK1, p21^Waf1/Cip1^, and INCA1, and downregulation of cyclin D1. Moreover, depletion of *CREB3L4* resulted in significantly decreased expression of a subset of androgen-receptor (AR) target genes, including *PSA, FKBP5, HPGD, KLK2*, and *KLK4*. We also demonstrated that CREB3L4 directly interacts with the AR, and increases the binding of AR to androgen response elements (AREs). We also identified a role for the unfolded protein response (and its surrogate, IRE1α), in activating *CREB3L4.* Cumulatively, we postulate that CREB3L4 expression is mediated by an AR-IRE1α axis, but is also directly regulated by AR-to-ARE binding. Thus, our study demonstrates that CREB3L4 plays a key role in PC cell proliferation, which is promoted by both AR and IRE1α.

Prostate cancer is one of the most commonly diagnosed cancers among men in industrialized nations, and is a leading cause of cancer-related deaths^1^. Proliferation of prostate cancer cells is known to depend on androgen receptor (AR) signaling^2^. The AR promotes the growth and regulates the activity of the normal prostate, and remains expressed in nearly all prostate tumors, even in recurrent androgen-independent tumors^3^,^4^. Since AR affects prostate cancer development through the regulation of not only transcription networks, but also genomic stability and DNA repair^5^, prostate cancer treatment relies on strategies targeting AR activity. Although androgen deprivation therapy (ADT) is initially effective in most cases, acquired resistance (termed castrate-resistant prostate cancer (CRPC)) typically develops, which is often characterized by acquired mutations in AR signal pathway genes^2^,^6^,^7^.

The *prostate-specific antigen (PSA*) gene is the best-characterized androgen-responsive gene in the prostate. Biochemical and genetic studies revealed that both the enhancer and the promoter of the *PSA* gene are androgen-responsive (independent of each other), but for the maximum expression of PSA, both its promoter and enhancer activities are necessary^8^.

It has been well shown that the endoplasmic reticulum (ER) plays a key role in normal prostate function, and various stimuli that cause ER stress could result in prostate pathologies, including benign prostate hyperplasia (BPH) and prostate cancer^9^. Indeed, there is a strong correlation between the occurrence of prostate cancer and the expression of ER stress protein markers, mainly via AR signal transduction^10^. In addition, activated AR directly binds the *inositol-requiring enzyme 1 (IRE1α*), as well as *x-box binding protein 1 (XBP1*). The latter two proteins target the ribosome-associated membrane protein 4 (*RAMP4*) and the *ER degradation-enhancing alpha-mannosidase-like 1 (EDEM1*) genes, in prostate cancer cells^11^. Indeed, AR and UPR gene expression are positively correlated in human prostate cancer patient samples^11^.

CREB3L4 (cyclic AMP-responsive element-binding protein 3-like 4, also called AIbZIP, Tisp40, or ATCE1) is an ER membrane-bound bZIP domain-containing transcription factor^12^. It was reported that *Creb3l4* is expressed in preadipocytes and functions as a gatekeeper, inhibiting adipogenesis^13^. *Creb3l4* disruption causes ER stress, formation of abnormal epididymal sperm nuclei, and caspase activation^12^, leading to apoptosis of meiotic and postmeiotic germ cells^14^. CREB3L4 is abundantly expressed in the prostate, and is much more highly expressed in cancerous than non-cancerous prostate cells^15^,^16^. Specifically, CREB3L4 is abundantly expressed in high-grade prostatic intraepithelial neoplasia (PIN), and all grades of adenocarcinomas, as compared to the normal prostate^15^. Thus, CREB3L4 could represent a potential biomarker for distinguishing benign from malignant prostatic cancer^17^. Although CREB3L4 expression is known to be regulated by androgen^15^, its role in prostate cancer progression and development is not well understood.

To this end, we explored a role for CREB3L4 in the androgen-dependent prostate cancer cell line, LNCaP. Our overall results demonstrate a role for CREB3L4 in modulating AR action, suggesting an interrelationship, and an AR-ER stress-CREB3L4 signaling axis, in prostate cancer cell proliferation.

## Results

### CREB3L4 is overexpressed in androgen-dependent LNCaP cells, and is further induced by androgen

**Figure 1:**
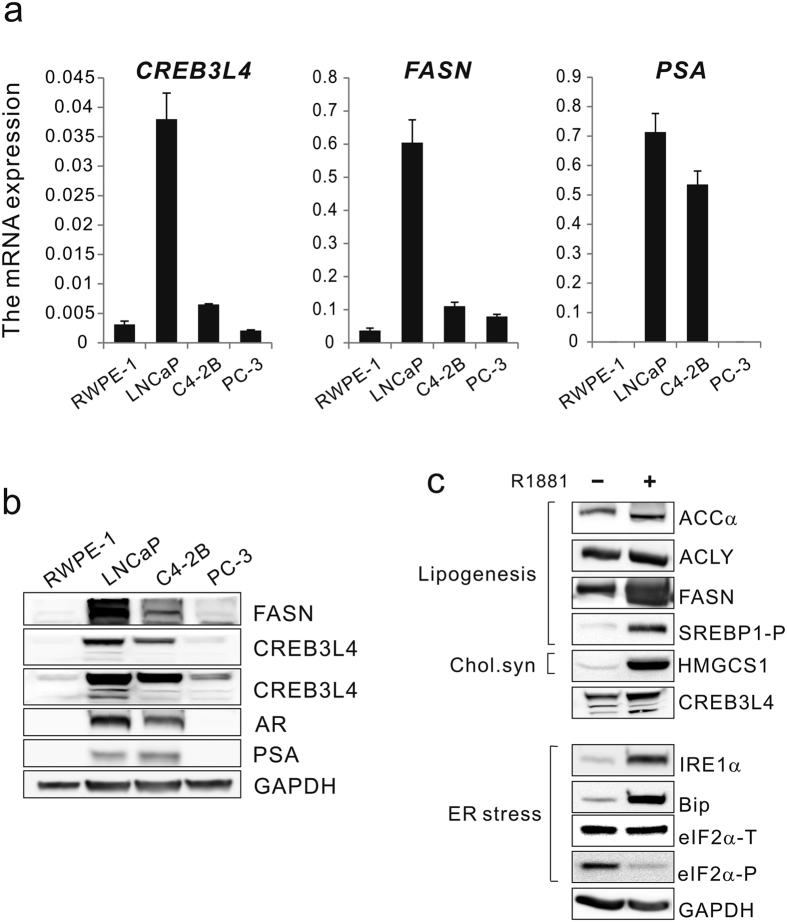
*CREB3L4* is overexpressed in androgen-dependent prostate cancer cell lines, and is induced by androgen. (**a**) Total RNA was extracted from cultured prostate cancer cell lines and real time qPCR performed. Levels of all mRNAs were normalized to those of *RPL19* mRNA. (**b**) Whole cell lysate protein (40 μg) of various prostate cancer cell lines was resolved by SDS/PAGE, and immunoblotted for the respective proteins indicated, with GAPDH intensity used as a loading control. (**c**) LNCaP cells were incubated for 1 day in the absence or presence of 10 nM R1881, in 1% CT-FBS medium, to optimize androgen effect(s). FASN, fatty acid synthase; PSA, prostate-specific antigen; ACCα, acetyl CoA carboxylase alpha; ACLY, acetyl-CoA lyase; GAPDH, glyceraldehyde 3-phosphate dehydrogenase; RPL19, ribosomal protein L19.

To explore the biological role of CREB3L4 in prostate cancer, we first observed the expression level of *CREB3L4* in prostate cancer cell lines, including the androgen-dependent prostate cancer (ADPC) cell line LNCaP, the androgen-insensitive metastatic subline C4-2B (derived from parental ADPC LNCaP cells), the androgen-independent prostate cancer (AIPC) cell line PC-3, and the nontumorigenic prostatic epithelial cell line RWPE-1. As shown in Fig. 1a, LNCaP cells expressed the highest levels of *CREB3L4*, fatty acid synthase (*FASN*), and *PSA*, as compared to the other cell lines. Along with its mRNA, CREB3L4 protein expression was higher in LNCaP than other cell lines (Fig. 1b). Western blot revealed that CREB3L4 expression was significantly increased by the synthetic androgen R1881, compared to untreated LNCaP cells (Fig.1c). These results support previous data showing the expression of genes related to fatty acid and cholesterol biosynthesis, and IRE1α, all significantly increased by R1881, whereas the protein kinase RNA-like endoplasmic reticulum kinase (PERK)-eukaryotic initiation factor 2 (eIF2α)-phosphorylation ER stress pathway, was suppressed by androgen^11^,^18^. From this background, we used LNCaP cells as an experimental model for studying androgen-dependent regulation of CREB3L4, with specific regard to prostate cancer cell proliferation.

### CREB3L4 is required for LNCaP cell proliferation

**Figure 2:**
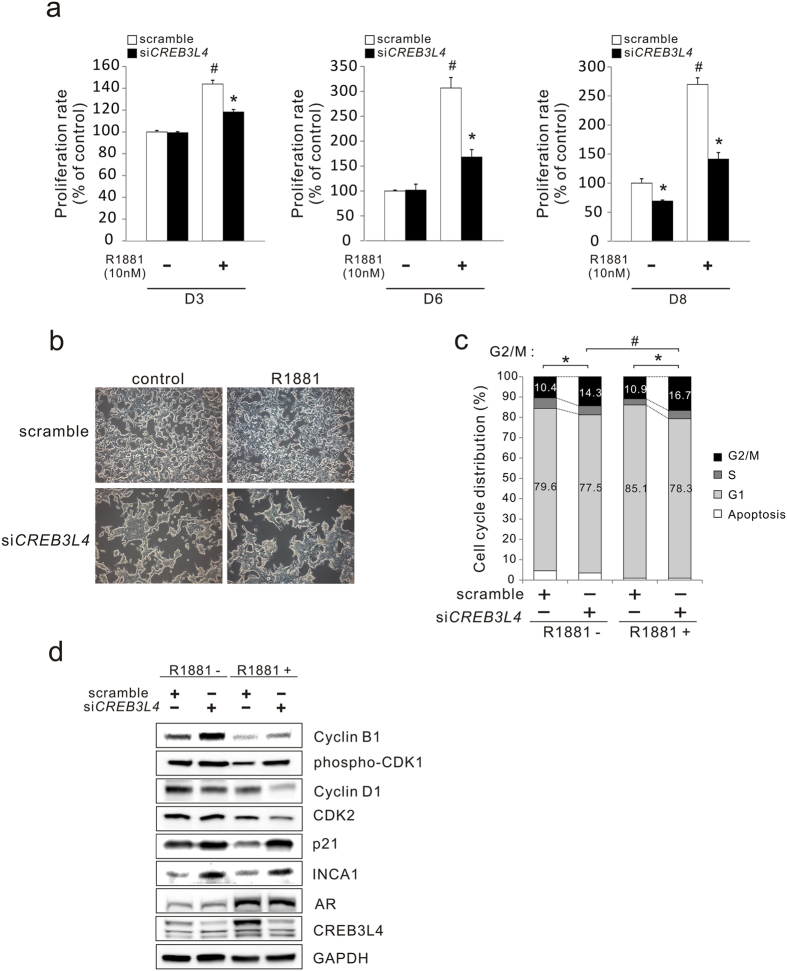
CREB3L4 regulates prostate cancer cell growth. (**a**) LNCaP cells were transfected with scrambled or si*CREB3L4* siRNAs. After 24 hr, the cells were treated with 10-nM R1881 or DMSO/EtOH control in 5% CT-FBS-containing medium, which was refreshed every 2 days. At 3/5/8 days after transfection, proliferation rates of the cells were measured by CCK-8 assay. (**b**) LNCaP cells were transfected with scrambled or si*CREB3L4*, in the presence or absence of R1881 (10-nM). At 48-hr after transfection, microscopic analysis of the cells was performed (x20). (**c**) Cell cycle profiles, of LNCaP cells transfected with *CREB3L4*-specific siRNA (si*CREB3L4*), in the presence or absence of 10-nM R1881 in 5% CT-FBS medium, were analyzed by propodium iodide (PI) staining and flow cytometry. As shown, there were significant differences in the G2/M population between scrambled versus si*CREB3L4*, in the absence/presence of R1881 (*P < 0.05), and si*CREB3L4* versus si*CREB3L4*, in the presence of R1881 (#*P* < 0.05). (**d**) LNCaP cells were transfected with scrambled or si*CREB3L4*. After 24 hr, the cells were treated with R1881 (10 nM) or DMSO/EtOH (control) in 5% CT-FBS-containing medium, for 24-hr. Whole cell lysate protein (40-μg) was resolved by SDS/PAGE, and immunoblotted for the respective proteins indicated, with GAPDH used as a loading control. Bars represent means ± S.E. (error bars), n = 3, *P < 0.05, scrambled versus si*CREB3L4*, in the absence/presence of R1881; #*P* < 0.05, scrambled versus scrambled, in the absence of R1881. DMSO, dimethyl sulfoxide; EtOH, ethyl alcohol; CT-FBS, charcoal treated-fetal bovine serum.

We next investigated whether CREB3L4 affects the growth of LNCaP cells. For this, we transfected si*CREB3L4*, and observed proliferation of cells for a period of 8 days (D3, D6, D8) in the presence or absence of R1881 (Fig. 2a). LNCaP transfection with si*CREB3L4* with or without R1881 inhibited proliferation at D3 by 20% (p < 0.05), D6 by 50% (p < 0.05), and D8 by 50% (p < 0.05), respectively. Specifically, from D3 to D8 after transfection, androgen-dependent proliferation of LNCaP cells was suppressed by si*CREB3L4* transfection (Fig. 2a). si*CREB3L4* also inhibited cell proliferation, even in the absence of androgen, at D8 (Fig. 2a). Microscopic observation also indicated that *CREB3L4* knockdown resulted in LNCaP growth inhibition (Fig. 2b). We observed decreased numbers of G1 phase cells, with increased numbers of G2/M-arrested cells, regardless of the presence of androgen (Fig. 2c), although androgen even further increased G2/M arrest, by suppressing CREB3L4. These findings further support a role for CREB3L4 in LNCaP cell proliferation. To confirm whether CREB3L4 affects cell cycle regulation in cell growth, we assessed expression of proteins involved in the cell cycle, especially G2/M arrest-related proteins (Fig. 2d). We found that suppression of *CREB3L4* resulted in the induction of cyclin B1, with repression of cyclin D1, causing mitotic clonal delay. Furthermore, expression of p21^Waf1/Cip1^ and INCA1 (inhibitor of cyclin-dependent kinase (CDK) interacting with cyclin A1), in complex with CDK2, respectively^19^, was increased by si*CREB3L4* (Fig. 2d). In addition, si*CREB3L4* increased phospho-CDK1 with decreased cyclin D1 and CDK2 in the presence of androgen, making it likely that CREB3L4 affects G2/M arrest. These data suggest that CREB3L4 affects the proliferation of LNCaP cells by inhibiting cell cycle transitions.

### CREB3L4 regulates AR-mediated transcription

**Figure 3:**
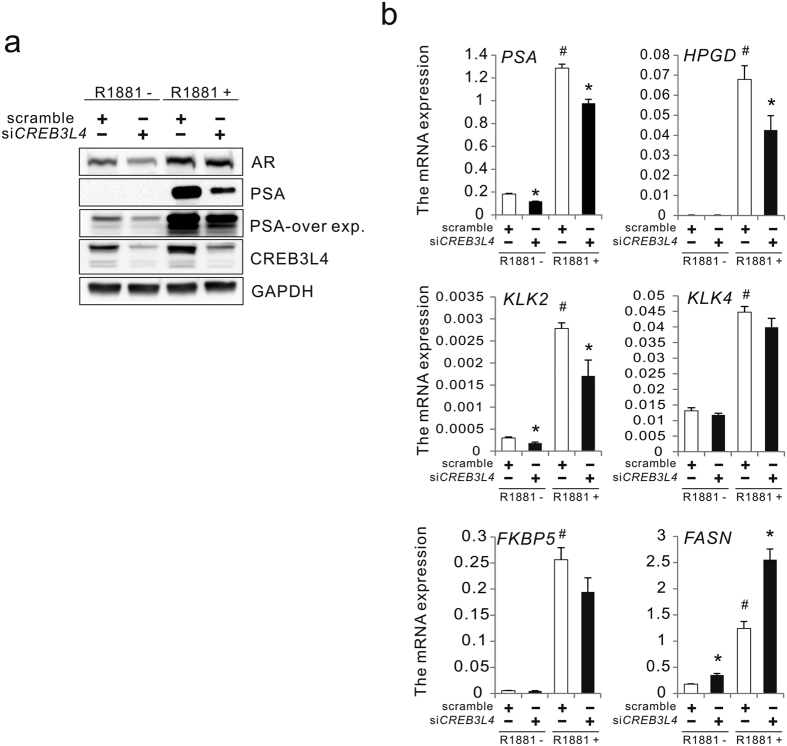
CREB3L4 regulates AR-mediated transcription. (**a**) LNCaP cells were transfected with scrambled or si*CREB3L4*. After 24 hr, the cells were treated with 10-nM R1881 or DMSO/EtOH control in 5% CT-FBS-containing medium, for 24 hr. Whole cell lysate protein (40 μg) was resolved by SDS/PAGE and immunoblotted for the respective proteins indicated, with GAPDH as a loading control. (**b**) Real-time PCR analysis showing the effect of si*CREB3L4* on the activation of AR target genes. LNCaP cells treated with si*CREB3L4* were harvested after treatment with 10-nM R1881. Total RNA was analyzed by real-time quantitative PCR, with the levels of all mRNAs normalized to those of *RPL19* mRNA, and expressed as folds-change. Statistical significance of differences between experimental groups was assessed by nonparametric Mann-Whitney test. Values were expressed as means ± S.E. (error bars), n = 3, *P < 0.05, scrambled versus si*CREB3L4*, in absence/presence of R1881; #*P* < 0.05, scrambled versus scrambled, in the absence of R1881. DMSO, dimethyl sulfoxide; EtOH, ethyl alcohol; CT-FBS, charcoal treated-fetal bovine serum.

Since AR plays a crucial role in prostate cancer cell proliferation, and is a common therapeutic target, we assessed possible roles for CREB3L4 in the AR signal cascade. Treatment of si*CREB3L4*-LNCaP cells with R1881 resulted in a significant reduction in protein levels of PSA, a well-known target of AR, even while AR expression was not changed (Fig. 3a). si*CREB3L4* also decreased PSA expression levels in the absence of androgen (Fig. 3a). To confirm the effect(s) of CREB3L4 on the expression of various AR-downstream genes, their expression was observed in androgen-treated cells expressing si*CREB3L4*. Knockdown of *CREB3L4* significantly decreased the expression of AR target genes, such as *PSA, HPGD, FKBP5, KLK2*, and *KLK4*, all of whose expression is tightly associated with prostate cell proliferation^20^ (Fig. 3b). These data suggest that CREB3L4 may act as a positive regulator of AR.

### CREB3L4 enhances AR activity through direct interaction with AR

**Figure 4:**
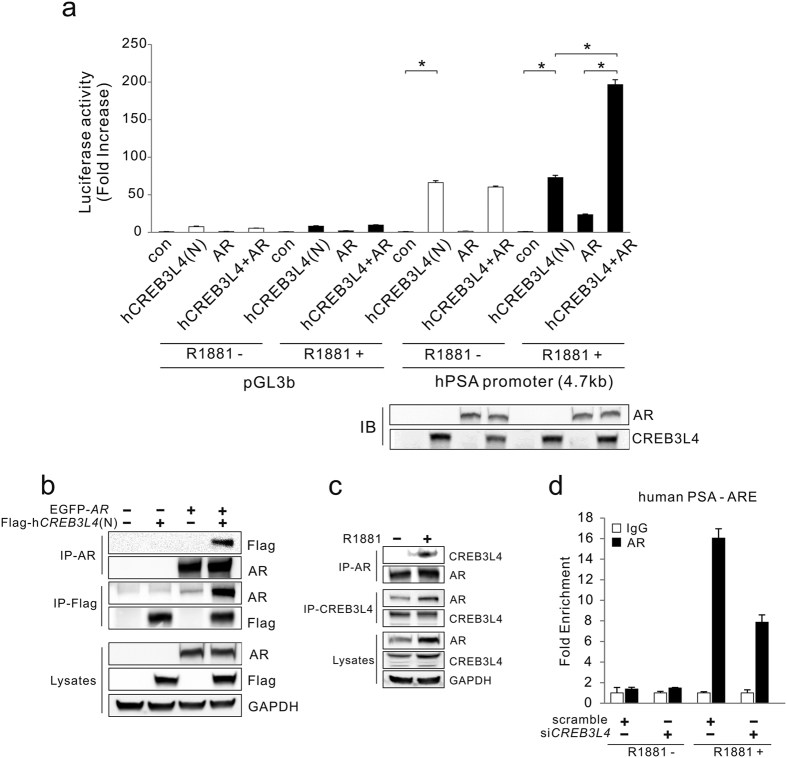
CREB3L4 enhances AR transactivation through interacting with AR. (**a**) Plasmids expressing *hCREB3L4N* (nuclear protein) and/or AR, under the *pGL3b* or human *PSA* gene promoters, were transfected into HEK293 cells, followed by treatment with or without 10-nM R1881. After 24 hr, cell lysates were subjected to luciferase assay. Luciferase activities were normalized to *Renilla* luciferase activities to adjust for transfection efficiency. Normalized activities are shown as means ± S.E. (error bars), n = 3, and expressed as folds-increase, relative to basal activity. (**b**) HEK293 cells were transfected with *AR*, and *CREB3L4* expression vectors, and proteins immunoprecipitated (IP’ed) using an anti-AR and anti-Flag for CREB3L4 antibodies. IP protein was then resolved by SDS/PAGE and immunoblotted for the respective proteins indicated. (**c**) Interaction of endogenous AR and CREB3L4 in LNCaP cells. Cells were treated with 10-nM R1881 for 24 hr. Endogenous AR and CREB3L4 protein from LNCaP cells were precipitated with anti-AR or anti-CREB3L4 antibodies, and the interaction between these proteins determined by co-IP. (**d**) ChIP assay performed in LNCaP cells transfected with si*CREB3L4*, with R1881 treatment. Normal IgG was used as a negative control for IP. AR response element (ARE) of *PSA* gene promoter was amplified to determine ChIP’ed DNA, which was normalized to total input DNA.

Then, a question arises. What is the role of CREB3L4 in the AR-dependent signaling pathway? To examine the relationship between CREB3L4 and AR, with regard to prostate cancer cell proliferation, we performed reporter assays using a *PSA* gene promoter construct. Those reporter assays showed that human nuclear form of *CREB3L4 (hCREB3L4N*) activated the *PSA* promoter activity by 50-fold, in the absence of androgen and AR (Fig. 4a). This data is consistent with our other results showing that PSA expression is suppressed in si*CREB3L4*-LNCaP cells in the absence of androgen (Fig. 3a). These data indicate that *PSA* gene expression could be directly regulated by CREB3L4.

In the presence of androgen, however, AR activated the *PSA* gene promoter reporter by 25-fold (Fig. 4a). However, AR-mediated transactivation of *PSA* promoter activity in the presence of androgen was significantly increased, by 200-fold, when the *hCREB3L4N* was cotransfected (Fig. 4a). This result suggests that *hCREB3L4N* upregulates AR-mediated transactivation, in a ligand-dependent manner. Consequently, we next explored the mechanism of how CREB3L4 activates AR signaling. We first examined the interaction between CREB3L4 and AR by co-immunoprecipitation (co-IP), showing that the two proteins interact with each other (Fig. 4b). Furthermore, the interaction between endogenous CREB3L4 and AR in LNCaP cells was also increased in the presence of androgen (Fig. 4c). To observe a possible role for CREB3L4 in AR-to-DNA recruitment, we performed chromatin immunoprecipitation (ChIP) assays. Androgen-induced AR recruitment to an androgen-response element (ARE), within the *PSA* gene promoter, was abolished in si*CREB3L4*-transfected cells (Fig. 4d). This result suggests that CREB3L4 interacts with AR, and assists AR recruitment to ARE. Taken together, these data suggest that not only can CREB3L4 upregulate the *PSA* gene AR-independently, it also enhances AR-mediated transactivation of its target genes by acting as an activator.

### Androgen-induced *CREB3L4* expression is regulated both directly, by AR, and indirectly, via IRE1α pathway signaling

**Figure 5:**
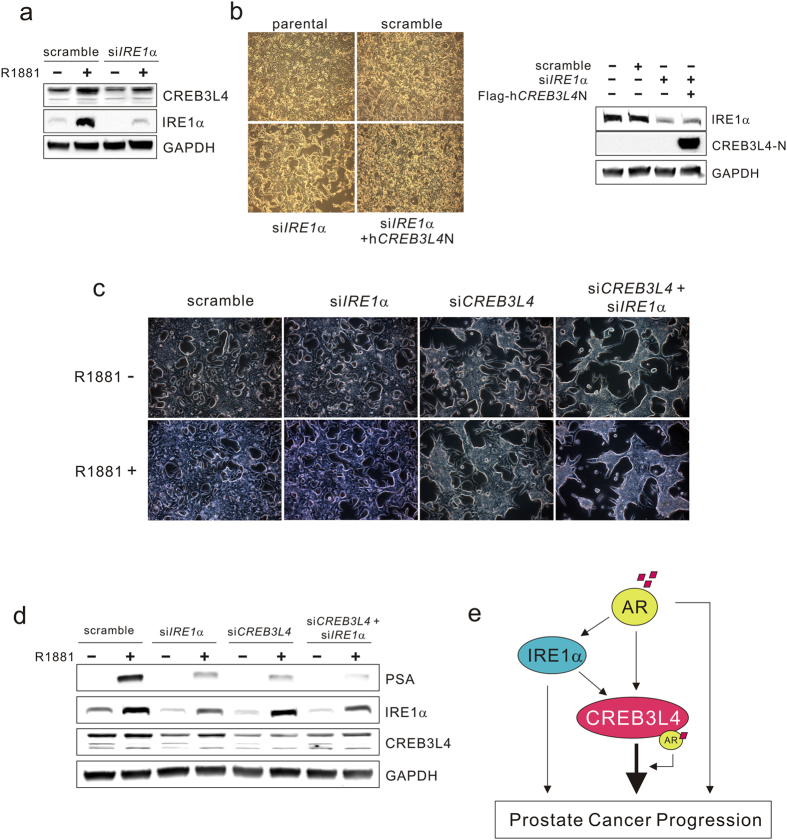
Androgen-induced CREB3L4 expression is regulated by IRE1α pathway. (**a**) LNCaP cells were transfected with scrambled or si*IRE1α*. After 24 hr, the cells were treated with 10-nM R1881 or DMSO/EtOH control in 5% CT-FBS-containing medium, for 24 hr. Whole cell lysates (40 μg) was resolved by SDS/PAGE and immunoblotted for the respective proteins indicated, with GAPDH used as a loading control. (**b**) LNCaP cells were transfected with scrambled or si*IRE1α* with *hCREBL4N* overexpression vector. 48 hr after transfection, microscopic analysis of the cells was performed (x10, upper panel). Western blot was performed to confirm the expression of IRE1α and CREB3L4 expression (lower panel). (**c**) LNCaP cells were transfected with si*IRE1α* and/or si*CREB3L4* in the presence or absence of R1881 (10-nM). At 48 hr after transfection, microscopic analysis of the cells was performed (x20) (**d**), and analyzed by western blot, to confirm the expression of PSA, IRE1α, and CREB3L4, with GAPDH as a loading control. (**e**) Proposed mechanism of action of CREB3L4 in prostate cancer progression.

We next observed that androgen promotes LNCaP cell proliferation by inducing CREB3L4 expression, which cooperatively upregulates AR target genes. Thus, we speculated whether CREB3L4 may also play a critical role in regulating prostate cancer cell proliferation, through interacting with AR. We first questioned the molecular mechanism of how *CREB3L4* gene expression is regulated in prostate cancer cells. It was reported that *CREB3L4* expression is increased by androgen^15^,^16^, which we confirmed in Fig. 1c. Recently, canonical unfolded protein response (UPR) pathways were also shown to be directly and divergently regulated by androgens in prostate cancer cells, through modulation of AR transactivation^11^, including that of IRE1α, with inhibition of the PERK signaling pathway^11^. In addition, full-length CREB3L4 is cleaved to its nuclear form, upon ER stress, in LNCaP cells^21^. From this basis, we assumed that androgen-induced *CREB3L4* expression could also be mediated through an AR-IRE1α signaling branch of the ER stress response. To test this hypothesis, we examined the relationship between IRE1α pathway signaling and CREB3L4 in AR-induced prostate cancer cell proliferation. These studies revealed that androgen-induced expression of *CREB3L4* was inhibited by depletion of *IRE1α* (Fig. 5a), which also significantly decreased LNCaP cell proliferation. Ectopic expression of *CREB3L4* in *IRE1α*-knockdown cells restored proliferation back to the level of control (i.e., non-transfected) LNCaP cells (Fig. 5b). Analogously, si*CREB3L4*-mediated inhibition of cell proliferation was further inhibited cell growth by si*IRE1α* (Fig. 5c). To examine the relationship between IRE1α and CREB3L4 in AR activity, we observed *PSA* expression levels following knockdown of *IRE1α* and/or *CREB3L4. PSA* levels were reduced by si*CREB3L4*, with even further reduction in cells cotransfected with si*IREα* (Fig. 5d). We next determined whether AR directly regulates *CREB3L4* gene expression, via CHIP assays. Using Vector NTI Suite, we found four putative androgen response elements (AREs) (inverted repeated (IR) sequence-IR3; AGAACANNNTGTTCT; ARE1~ARE4) within the 3 kb region of the human *CREB3L4* promoter (Supplementary Fig. 1a). Moreover, as might be expected, we observed that R1881 increases endogenous AR recruitment to the putative ARE4 region of the *CREB3L4* promoter (Supplementary Fig. 1b). These results suggest that androgen-induced *CREB3L4* gene expression is in part regulated by AR directly. Furthermore, activation of ER stress response signals by AR contributes to *CREB3L4* upregulation, resulting in activation of the androgen-dependent transcriptional cascades.

## Discussion

CREB3L4 is highly expressed in the prostate gland, and upregulated even further in prostate cancer^15^,^22^. Indeed, CREB3L4 is now being evaluated as a potential marker for prostatic diseases, based especially on its ability to discriminate between benign and malignant prostate tumors^17^. However, the biological role of CREB3L4 in prostate cancer cell proliferation previously remained unknown. In this study, we found that CREB3L4 is essential for prostate cancer cell proliferation, through its modulation of AR activity, which reciprocally, is upstream of *CREB3L4* expression (perhaps suggesting some type of positive feedback). We also showed that CREB3L4 directly associates with AR, to enhance AR-induced transactivation of its downstream genes, in prostate cancer cells. Furthermore, we observed that AR-induced CREB3L4 expression is also mediated by IRE1α, ER stress response signaling. Our data thus suggest that the novel link between AR-CREB3L4 and the IRE1α pathway is an axis critical for driving prostate cancer progression (Fig. 5e).

The unfolded protein response (UPR) has critical roles in development and normal physiology, as well as in pathological states such as cancer^23^. The IRE1α-XBP1 pathway is known to be essential for tumor survival^24^, and loss of XBP1 sensitized cancer cells to death from oxidative stress^25^. Specifically, androgens induce a UPR response in prostate cancer cells, by activating the IRE1α-XBP1 signaling branch, to regulate growth and survival of prostate cancer cells^11^. XBP1 expression is also increased in clinical prostate cancer specimens^11^. Since CREB3L4 is upregulated by ER stress^14^, we assumed that androgen-induced CREB3L4 expression is mediated through the IRE1α pathway. We confirmed that AR-induced CREB3L4 expression is abolished through knockdown of *IRE1α* (Fig. 5a). In addition, we observed that CREB3L4 promoter activity is significantly increased by overexpressing *hXBP1*, a transcription factor gene downstream of the IRE1α pathway (Supplementary Fig. 1c). These results suggest that androgen-induced *CREB3L4* gene expression is in part regulated by AR directly (due to physical interaction with the *CREB3L4* promoter), and in part mediated indirectly, via an IRE1α pathway. Thus, we posit that CREB3L4 is an essential mediator of AR-IRE1α-induced prostate cancer progression.

*CREB3L4* knockdown also increased the number of cells in G2/M, and upregulated p21^Waf1/Cip1^ and INCA1, both inhibitors CDK, regardless of the presence of androgen (Fig. 2c,d). However, phosphorylation of CDK1, after si*CREB3L4* transfection, was further increased in the presence, but not absence, of androgen (Fig. 2d). Moreover, CDK1 phosphorylation coincided with a small, but statistically significant, G2/M arrest in si*CREB3L4*-transfected and R1881-treated cells, vs. androgen-untreated si*CREB3L4* cells. This result shows that androgen could play an additive role in si*CREB3L4*-mediated arrest of G2/M phase prostate cancer cells. Indeed, we observed that si*CREB3L4* transfection resulted in significant growth inhibition of LNCaP cells, in the presence of R1881, at D6 and D8 (Fig. 2a). This result suggests that androgen-induced CREB3L4 significantly increases cell proliferation.

There is a question whether CREB3L4 can even function in the absence of androgen. To address this, we showed that cell proliferation by D8, even in the absence of R1881, was decreased by si*CREB3L4* (Fig. 2a,b). CREB3L4 also increased *PSA* expression independently of AR (Fig. 4a), and had a greater activating effect on the *PSA* promoter, compared to that of AR alone (Fig. 4a). Furthermore, transfection of si*CREB3L4* decreased PSA protein levels in the absence of androgen (Fig. 3a). We also observed *IRE1α* downregulation, by *CREB3L4* knockdown, in the absence of androgen (Fig. 5d). Thus, it is possible that *IRE1α* is also regulated by CREB3L4, similar to *PSA*. Therefore, we could not exclude androgen-independent effects of CREB3L4 on LNCaP cell proliferation. However, CREB3L4 had a more robust effect in the androgen-dependent state, compared to the androgen-independent state (Fig. 2a). We speculate that regulation of the AR-IRE1α-CREB3L4 pathway in the presence of androgen, may maximize the effect of androgen on proliferation of prostate cancer cells.

How does CREB3L4 regulate androgen-dependent cancer cell proliferation? We showed here that *CREB3L4* knockdown resulted in decreased androgen-induced *PSA* expression, without affecting AR expression (Fig. 3a). Besides, *CREB3L4* depletion decreased AR binding to an ARE within the *PSA* gene promoter (Fig. 4d). This indicates that CREB3L4 promotes AR binding to AREs, and transactivation of the associated target genes, by directly interacting with AR. However, we could not confirm recruitment of CREB3L4 to the ARE within the *PSA* gene promoter, nor binding to an unfolded protein response element (data not shown), in contrast to another study^26^. Thus, it yet remains necessary to identify CREB3L4-binding sites, within gene promoters, to better understand its regulation of *PSA* and other AR-target genes. We assume that independently bound CREB3L4 may interact with AR (bound to its ARE) on the *PSA* promoter, presumably by bending, “looping,” or some other unknown mechanism. Hence, more study is required to understand CREB3L4 activation of *PSA*, or other downstream genes, in a manner that is synergistic with AR.

Previous reports have demonstrated that the enzymes involved in fatty acid synthesis and cholesterol synthesis are upregulated in prostate cancer cells by androgens^27^,^28^. Thus, it was surprising to note that the expression of *FASN* was increased in si*CREB3L4* knockdown cells. The mechanism of downregulation of *FASN* by the AR-CREB3L4 axis may be different from that of other AR target genes. Distinct cofactor complexes with CREB3L4/AR may occur in its regulation of lipogenesis-related genes. Thus, the molecular mechanism of this distinct regulation needs further study.

Collectively, we demonstrated that CREB3L4 is required for proliferation of prostate cancer cells, and that CREB3L4 is a crucial activator of AR function. Indeed, CREB3L4 directly interacts with, and facilitates, AR recruitment to the AREs of AR target genes, maximizing their expression, (e.g., *PSA*) (Fig. 3b). Additionally, we demonstrated that androgen-induced *CREB3L4* expression is in part regulated by AR directly, and in part indirectly, by IRE1*α* signaling, suggesting that a distinct AR-ER stress-CREB3L4 regulatory axis also plays a role in prostate cancer proliferation. A schematic of the mechanism of action of CREB3L4, in facilitating prostate cancer proliferation, is shown in Fig. 5e. In summary, our findings demonstrate that CREB3L4 plays a key role in prostate cancer cell proliferation, justifying its further study as a possible prostate cancer biomarker and therapeutic target.

## Methods

### Cell culture and Transient Transfection Assay

HEK293 (embryonic kidney) and PC3 (androgen-independent prostate cancer cells) were obtained from ATCC (Manassas, VA, USA) and grown in Dulbecco’s modified Eagle’s medium (Hyclone, Logan, UT, USA), supplemented with 10% (v/v) fetal bovine serum, 100 units/ml penicillin, and 100-μg/ml streptomycin. LNCaP (an androgen-dependent human prostate cancer cell line; ATCC number CRL1740) and RWPE-1 (nontumorigenic, prostatic epithelial cell line; ATCC number CRL11609) cells were grown at the Roswell Park Memorial Institute (RPMI, Buffalo, NY, USA) in medium 1640, supplemented with 10% FBS, and penicillin/streptomycin. C4-2B cell lines were supplied as a kind gift from Dr. H.G Yoon^29^. LNCaP cells were maintained in 10% FBS-containing media, and for all experiments, this media was replaced with 5% charcoal treated (CT)-FBS media to selectively remove hormones, growth factors, and steroids the day before observing the effects of R1881. Transient transfection and luciferase assays were performed using Fugene HD reagent (Promega, Madison, WI, USA) and a Dual luciferase assay kit (Promega). Luciferase activities were normalized to Renilla firefly activities, to adjust for transfection efficiency. Normalized luciferase activities are shown as means ± S.E. Data are expressed as fold-increases, relative to the basal activity of the reporters, in the absence of overexpression vectors.

### Flow cytometry analysis

siRNA-transfected LNCaP cells were harvested by trypsinization, and fixed in ice-cold 70% ethanol overnight. The cells were then washed twice with cold PBS, and incubated for 30 min at room temperature in 1-mL PBS containing 50 μg/ml propidium iodide (Invitrogen) and 50 μg/ml RNaseA (Sigma, St. Louis, MO, USA). The stained samples were then analyzed with a LSR-II flow cytometer (Becton Dickinson, Franklin Lakes, NY, USA).

### Cell viability assay

siRNA-transfected LNCaP cells were trypsinized and seeded in 5% CT-FBS-containing media, in the presence/absence of 10 nM R1881, in 96-well plates (7.5 × 10^3^ cells/well). Media containing 5% CT-FBS, with 10 nM R1881 or DMSO/EtOH (control), was changed every 2 days. At D3/D6/D8 after transfection, cell viability was measured, using the Cell Counting Kit-8 (Sigma) according to the manufacturer’s instructions. Briefly, 10-μl of CCK-8 solution was added to each well, and plates incubated for 4 hr, followed by measurement of absorbance at 450 nm, using a microplate reader.

### Total RNA isolation and quantitative real-time PCR

Total RNA was isolated from LNCaP/PC3/RWPE1/C4-2B cells using TRIzol reagent (Invitrogen, Carlsbad, CA, USA), according to the manufacturer’s instructions. This RNA was used to generate cDNA using the GoScript Reverse Transcription System (Promega). Quantitative real-time PCR (qPCR) was performed using the Step One Real-Time PCR Systems instrumentation and software (Applied Biosystems, Foster City, CA, USA), according to the manufacturer’s protocol. The relative amount of mRNA in each sample was normalized to that of the gene *RPL19*. Primers used in PCR were as follows: *CREB3L4*-f, 5′-GGCCTTCAAGAGAGTGAGCCTG-3′; *CREB3L4*-r, f, 5′-AACTGGGCAGGATGATGAGAGC-3′; *RPL19*-f, 5′-CACATCCACAAGCTGAAGGCAGAC-3′; *RPL19*-r, 5′-CGTGCTTCCTTGGTCTTAGACCTG-3′. The primers, *FASN, PSA, HPGD, KLK2, KLK4*, and *FKBP5*, were used for the real time PCR^18^,^20^.

### Western blotting

Proteins from LNCaP/PC3/RWPE1/C42B cells were isolated using RIPA solution (Thermo Scientific, Rockford, IL, USA) containing appropriate protease inhibitors. Protein concentration was determined using the BCA protein assay (Thermo Scientific), and 40 μg were subjected to sodium dodecyl sulfate - polyacrylamide gel electrophoresis (SDS-PAGE) and transferred to nitrocellulose membranes (Whatman, Dassel, Germany). Membranes were blocked with 5% non-fat milk and incubated with the following primary antibodies: anti-CREB3L4 (AT1618a, Abgent Inc., San Diego, CA, USA), anti-INCA1 (sc-243077) (Santa Cruz Biotechnology Inc., Santa Cruz, CA, USA), anti-AR (#5153s), anti-PSA (#2475s), anti-GAPDH (#2118), anti-IRE1α (#3294s), anti-Bip (#3711p), anti-eIF2α-total (#2103s), anti-eIF2α-phospho (#3597s), anti-cyclin B1 (#12231), anti-phospho CDK1 (#4539), anti-cyclin D1 (#2922), anti-CDK2 (#2546), anti-p21^Waf1/Cip1^ (#2947) (all from Cell Signaling, St. Louis, MO, USA), anti-HMGCS1 (ab194971, Abcam, Cambridge, MA, USA), and anti-Flag (F3165, Sigma). Antibodies for FAS, ACCα, ACLY, and SREBP1 were kindly provided by Dr. K.S. Kim^18^. Membranes were then incubated with horseradish peroxidase-conjugated-anti-mouse or -anti-rabbit goat secondary antibodies (Thermo Scientific), at a 1:4000 dilution, in 5% non-fat milk in PBST buffer (137 mM NaCl, 2.7 mM KCl, 10 mM Na_2_HPO_4_, 2 mM KH_2_PO_4_, 0.1% Tween-20), for 1 h at room temperature. Antibody-bound proteins were visualized using an enhanced chemiluminescence detection system (West Pico & West Dura, Thermo Scientific). The protein bands were also detected using a Fujifilm LAS-3000 Imager (FUJIFILM Corporation, Tokyo, Japan).

### Chromatin immunoprecipitation (ChIP) assay

ChIP experiments were carried out according to the standard protocol (Merck KGaA, Darmstadt, Germany). LNCaP cells were plated in 15-cm tissue culture plates and transfected with si*CREB3L4* siRNA, combined with 10 nM R1881 treatment. Cell lysates were then incubated for 15 hr at 4 °C with 5-μg anti-AR (#5153, Cell Signaling) or normal rabbit IgG (sc-2027, SantaCruz Biotechnology) antibodies. After reversal of crosslinking, immunoprecipitated DNA was quantified by qPCR. All reactions were normalized relative to total input to account for chromatin sample preparation differences (ΔC_t_), determined as the difference between the α-AR IP sample (ΔC_t [AR]_) and α-IgG IP sample (ΔC_t [IgG]_) for fold enrichment. Relative expression was determined by the method ΔΔC_t [AR - IgG]_ = ΔC_t [AR]_ - ΔC_t [IgG]_, and fold-change in occupancy = 2^(−ΔΔCt[AR-IgG])^^30^. The primer used for PCR of the AR promoter binding region (distal ARE) of the *PSA* gene is described in ref. 31. Primers sequence for PCR of four putative AREs were as follows: ARE1-f, 5′-AACCTGGATTCTGGTCCAAGTTCTA-3′; ARE1-r, 5′-AACTCCTGACCTCGTCATCTGC-3′; ARE2-f, 5′-TCAAATCATCTCTGCACATACA-3′; ARE2-r, AACCAAGATTTGGATGCTTCAG-3′; ARE3-f, 5′-GGAGCTTGCAGTGAGCCGAGATC-3′; ARE3-r, 5′-ACTGCTACTAATTATCCTTATGAAG-3′; ARE4-f, 5′-TGCATGGAACCGTGATCGCACCAC-3′; ARE4-r, 5′-AAGCTGAAGACTTAGGTTTCGGAG-3′.

### Immunoprecipitation assay

Constructs expressing Flag-tagged *hCREB3L4N* and GFP-tagged *AR* were cotransfected into HEK293 cells using FuGENE HP transfection reagent (Promega). To confirm the endogenous interaction between CREB3L4 and AR, LNCaP cells were treated with 10-nM R1881 for 48 hr, harvested, and lysed in cold cell lysis buffer (50-mM Tris [pH7.4], 150-mM NaCl, 0.2% Triton X-100, 0.3% NP-40), containing appropriate protease inhibitors. Whole cell lysates (1000 μg) were precleared, with protein G-agarose (#05015952001, Roche, Mannheim, Germany), followed by incubation with anti-AR, anti-CREB3L4, or anti-Flag antibodies, for 16 h with protein G-agarose, for 2 h at 4 °C. After centrifugation, the protein G-agarose pellets were washed several times with washing buffer (1/3 dilution of cell lysis buffer in PBS) and resuspended in sample buffer, before being subjected to SDS-PAGE.

### Small interfering RNA (siRNA)

RNA oligonucleotides for human *CREB3L4* (forward, 5′-GCUAGAUCAGUGGAGCCCAGCAUUU-3′) (Invitrogen) were synthesized. For silencing *IRE1α*, siRNA against *IRE1α* (Santa Cruz, sc-40705) was used. Medium GC Duplex was used as a negative control (Thermo Scientific, # 12935300). Each siRNA (40 nM) was transfected into appropriate experimental sets of LNCaP cells, using Lipofectamine RNA iMAX (Invitrogen) for at least 48 h, followed by cell lysis for RNA and protein preparation.

### Construction of plasmids

Expression constructs encoding the nuclear form of *hCREB3L4* and hXBPs were PCR-amplified and cloned directly into the pcDNA3.1-Flag2 vector. The construct pEGFP-C1-AR was purchased from Addgene (Cambridge, MA, USA). The human PSA promoter, covering about 4.7 kb, was a generous gift from Dr. K.S. Kim (Yonsei Univ., South Korea). The mouse *Creb3l4* gene promoter covering a −2500/+209 region was PCR-amplified and cloned directly into the pGL4b luciferase reporter vector.

## Additional Information

**How to cite this article:** Kim, T.-H. *et al*. The role of CREB3L4 in the proliferation of prostate cancer cells. *Sci. Rep.* **7**, 45300; doi: 10.1038/srep45300 (2017).

**Publisher's note:** Springer Nature remains neutral with regard to jurisdictional claims in published maps and institutional affiliations.

## Supplementary Material

Supplementary Information
