# Supplementary material for: The role of CREB3L4 in the proliferation of prostate cancer cells
Source: Sci Rep. 2017 Mar 24;7:45300. doi: 10.1038/srep45300 (PMC5364418; doi:10.1038/srep45300)
Supplement: Supplementary Information [file srep45300-s1.pdf]

Supplementary Information

The role of CREB3L4 in the proliferation of prostate cancer cells

Tae-Hyun Kim[1], Joo-Man Park[1], Mi-Young Kim[1], Yong-Ho Ahn[1, 2, *]

[1]Department of Biochemistry and Molecular Biology, Yonsei University College of Medicine, Seoul 120-752, Republic of Korea

[2]Brain Korea 21 PLUS Project for Medical Sciences, Yonsei University College of Medicine, Seoul 120-752, Republic of Korea

* Address correspondence to: Yong-Ho Ahn, M.D., Ph.D. ;

Dept. of Biochemistry and Molecular Biology,

Yonsei University College of Medicine,

50-1 Yonsei-ro, Seodaemun-gu, Seoul 120-752, Republic of Korea.

Tel: +82-2-2228-0835, Fax: +82-2-312-5041,

E-mail: yha111@yuhs.ac; yha111@hanmail.net

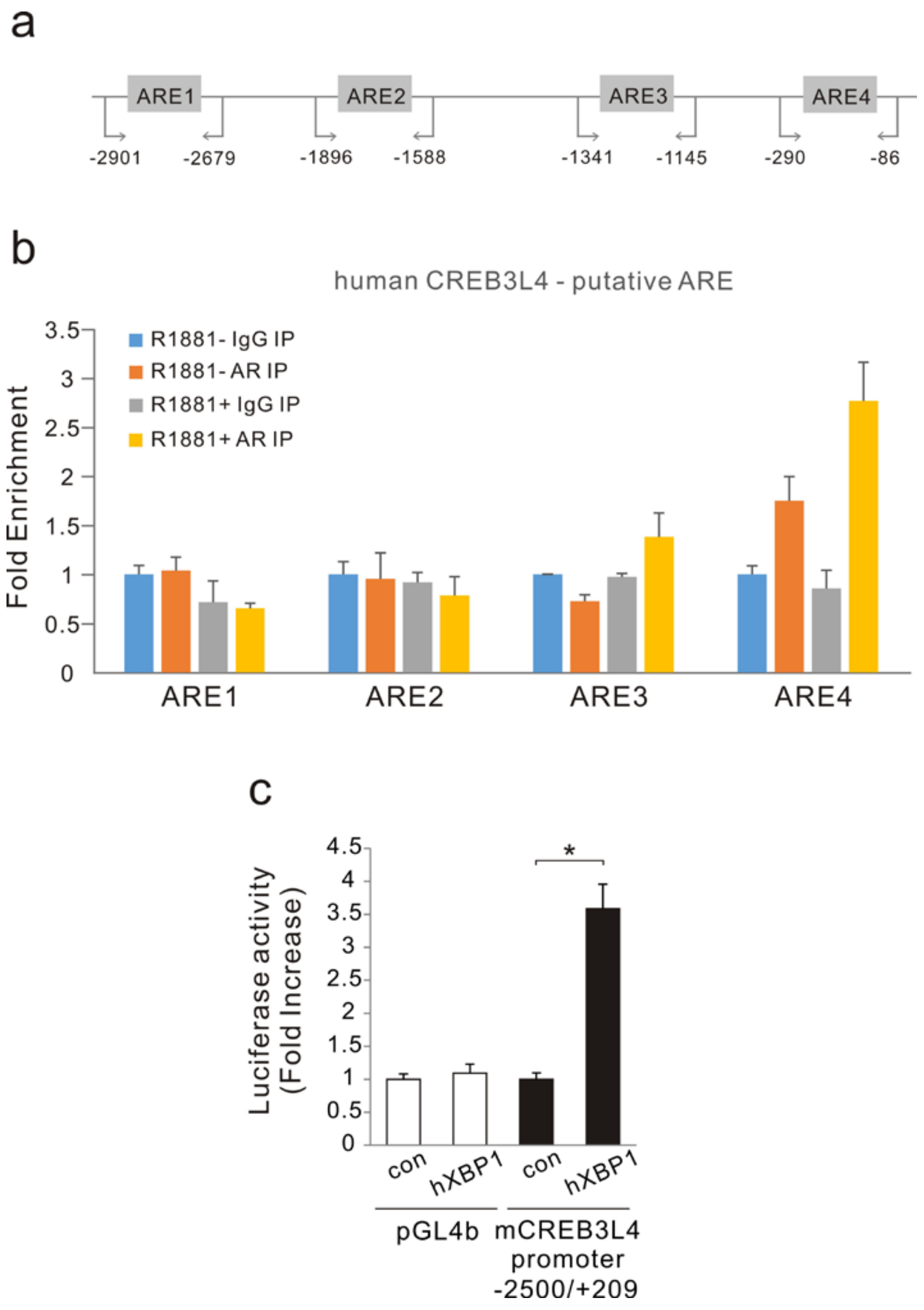

Supplementary Figure 1. Effect of AR and XBP1 on *CREB3L4* promoter activity. (a) Putative androgen response element (ARE) within 3kb of human *CREB3L4* promoter. (b) Chromatin immunoprecipitation (ChIP) assays were performed on LNCaP cells treated with synthetic androgen (R1881). Normal IgG was used as a negative control for ChIP. The ARE of the *CREB3L4* gene promoter was amplified, following ChIP pulldown, to determine AR-to-DNA binding, which was normalized to total input DNA. Error bars represent means ± S.E. (c) Luciferase activity of mouse *Creb3l4* gene promoter covering a -2500/+209 region. The human spliced isoform of XBP1 was transfected with mouse *Creb3l4* gene promoter reporter or pGL4b in HEK293 human kidney cells. Luciferase activity was normalized to *Renilla* luciferase activity, to adjust for transfection efficiency. Normalized activities are shown as means ±S.E. (error bars), n = 3, and reporter activities expressed as folds-increase, relative to basal activity. *$p<0.05$
